# Predicting response to topical non-steroidal anti-inflammatory drugs in osteoarthritis: an individual patient data meta-analysis of randomized controlled trials

**DOI:** 10.1093/rheumatology/keaa113

**Published:** 2020-04-10

**Authors:** Monica S M Persson, Joanne Stocks, Gyula Varadi, Mohammad Hashem Hashempur, Marienke van Middelkoop, Sita Bierma-Zeinstra, David A Walsh, Michael Doherty, Weiya Zhang

**Affiliations:** k1 Academic Rheumatology, School of Medicine, University of Nottingham, Nottingham, UK; k2 BioPhysics Pharma, Beverly, MA, USA; k3 Department of Traditional Persian Medicine, Fasa University of Medical Sciences, Fasa, Iran; k4 Department of General Practice, University Medical Center, Erasmus Medical Center, Rotterdam, The Netherlands

**Keywords:** osteoarthritis, topical NSAIDs, IPD meta-analysis, predictors

## Abstract

**Objectives:**

To identify predictors of the specific (difference between treatment and placebo) and overall (change from baseline in treatment arm) treatment effects of topical NSAIDs in OA.

**Methods:**

Randomized controlled trials (RCTs) of topical NSAIDs in OA were identified through systematic literature searching and inquiry to pharmaceutical companies. The raw, de-identified data were analysed in one-stage individual patient data meta-analysis (IPD-MA). Negative values for treatment effects (0–100 scale) indicate pain reduction.

**Results:**

Of 63 eligible RCTs, 15 provided IPD (*n* = 1951 on topical NSAID), including 11 placebo-controlled RCTs (*n* = 1587 on topical NSAIDs, 1553 on placebo). Seven potential predictors of response were examined. Topical NSAIDs were superior to placebo [−6 (95% CI −9, −4)], with a small, but statistically significant greater effect in women than men [difference −4 (95% CI −8, −1)]. The overall treatment effect was 4-fold larger than the specific effect [−25 (95% CI −31, −19)] and increased with greater baseline pain severity (*P* < 0.001). No differences in efficacy were observed for age, BMI, features of inflammation, duration of complaints or radiographic OA severity.

**Conclusion:**

Topical NSAIDs are effective for OA pain relief. Greater overall pain relief in individuals with more baseline pain might be due to contextual and non-specific effects, including regression to the mean. Additional factors that have been linked either mechanistically or through empirical evidence to outcomes should be selected for inclusion across future RCTs in order to facilitate the identification of response predictors through IPD-MA.

## Introduction

Pain is an important and distressing feature of OA and the most common reason for OA-related visits to primary care [1]. Topical NSAIDs are recommended treatments [2], which have been shown to be superior to placebo in randomized controlled trials (RCTs) [3]. However, little is known regarding any patient-level predictors of response to topical NSAIDs. Patient-level predictors of response could improve clinical decision-making by guiding optimal treatment selection for the individual patient at the time of assessment (precision medicine).

Treatment effects can be defined as specific treatment effects (i.e. the difference between treatment and placebo, resulting from the biological effect of the treatment itself) and overall treatment effects (i.e. the total improvement from baseline, which includes the specific effect, contextual effects from receiving a treatment and non-specific effects such as temporal variation in symptom severity) [4]. While the former is useful in demonstrating the potential efficacy of a new treatment, the latter explains the total benefits that an individual patient may obtain from a treatment in clinical practice [5]. RCTs are mainly powered to show benefit over a comparator (either placebo or active) and, on their own, usually have insufficient power for robust subgroup analysis or analysis of potential response predictors. Individual patient data meta-analyses (IPD-MAs) involve the re-examination of raw, de-identified patient-level data from relevant RCTs, thus increasing the power relative to primary studies and overcoming widely recognized limitations of aggregate data met-analyses (AD-MAs) [6]. Therefore IPD-MAs may identify clinically important response predictors, which could prove useful in shared decision-making with respect to treatment selection based on individual patient characteristics. The aim of the present study was to examine predictors of the specific and overall treatment effect of topical NSAIDs in OA using an IPD-MA of RCTs.

## Methods

### Protocol and registration

The study is part of body of work, the protocol of which is published [7] and available on PROSPERO (2016; CRD42016035254). We were unable to conduct an IPD-MA for topical capsaicin as pre-specified [7], as none of the data custodians were willing or able to contribute data (10 eligible RCTs). The present work therefore examines only topical NSAIDs in OA.

### Study selection

The process for study selection is available in the protocol [7] and is briefly described below. RCTs comparing topical NSAIDs to any active or placebo comparator in participants with OA were eligible [7]. RCTs had to have a minimum duration of 1 week and report pain outcomes. Trials were identified through systematic literature searches (to November 2015) of six databases (MEDLINE, Embase, Allied and Complementary Medicine Database, Cumulative Index to Nursing and Allied Health Literature, Web of Science and Cochrane Library) and scrutiny of the reference lists of included publications and MAs in the area (Supplementary material, section Example literature search strategy, available at *Rheumatology* online). In addition, unpublished RCTs were sought from pharmaceutical companies that manufacture topical NSAIDs for sale in the UK or that have registered trials for the medications. Companies were identified via the British National Formulary or electronic Medicines Compendium. Trial registrations were searched via clinicaltrials.gov and clinicaltrialsregister.eu. Approval from a research ethics committee was not required, as the work involved analysis of de-identified data and no new data collection was undertaken.

### AD

AD extraction and risk of bias assessment were conducted independently by two authors (M.S.M.P. and J.S.). Data extracted included publication information, trial design, participant demographics, interventions and pain outcome data. Risk-of-bias assessment was conducted using a modified Cochrane Risk of Bias tool [7, 8]. AD were used to determine whether the captured IPD were representative of the published evidence base.

### IPD collection and management

The first or corresponding author of all eligible trials was contacted using a standardized e-mail, personalized to include the author name and study details. Where no response was received, additional attempts to contact data custodians were made by sending two reminder e-mails, contacting via letter and telephone, contacting all other publication authors, contacting the institution where the research was conducted and reaching out to the trial funder or sponsor. Unless contact details were unavailable, all additional approaches were implemented for the studies until a definitive response (accepting or declining collaboration) was received or data collection was closed.

Data custodians that expressed an interest in collaboration were asked to sign a data transfer agreement developed by the OA Trial Bank [9] or the University of Nottingham outlining the terms for collaboration and transfer of data.

Collaborators were given the option to share the whole anonymized dataset or only the variables required for analysis in the IPD-MA. Baseline variables sought were pre-specified and are listed in Table 1. Baseline and follow-up pain data at all durations of assessment were collected. Where multiple assessments of pain were available, visual analogue scale (VAS) global pain scores were prioritized [3]. If unavailable, categorical global pain scores, VAS pain during activity or disease-specific composite tools were used instead [3]. The hierarchical ordering of outcomes was specified *a priori* [7].

**Table 1:** Baseline data sought from data custodians

Participant ID
Date of randomization/inclusion
Age or date of birth
Sex
Weight
Height
BMI
Duration of complaints
Signs of inflammation—clinical (e.g. effusion) or biochemical (e.g. ESR, CRP)
Nature of pain (dull/neuropathic)
Indicators of central sensitization
Psychological assessments (e.g. depression, anxiety, catastrophizing)
Index joint
Radiographic OA severity

On receiving the IPD, an initial screen of the data was conducted to ensure that IPD for all randomized participants were received. Any discrepancies were discussed. A consistent approach to coding, variable labelling, standardization of variables and dichotomizing continuous variables was established (Supplementary Table S1, available at *Rheumatology* online). A study identifier was given to each trial and participants retained their original study-specific participant identifier. Pain scores were standardized to a 0–100 scale within each study [10].

Analyses were based on two treatment effects: specific and overall treatment effects. Potential predictors of both specific and overall treatment effects were examined. Person-level characteristics investigated were those pre-specified in Table 1. These were chosen *a priori* as recognized peripheral and central risk factors of OA or OA pain and were examined if available in more than one RCT. Treatment effects are presented as the difference between the treatment and placebo groups (specific effect) or within the treatment arm (overall effect) on a 0–100 scale for pain. Only placebo-controlled RCTs were used to examine the specific treatment effect, while all placebo- and active-controlled RCTs were analysed for the overall treatment effect.

### Statistical analysis

#### AD-MA

Published placebo-controlled RCTs were combined in a random effects AD-MA for the specific and overall treatment effects. Effect sizes (ESs) were calculated using Hedges’ *g* [11]. ESs were back-translated to a 0–10 cm VAS [12] and multiplied by 10 for direct comparison with the IPD-MA. As the focus of the work was to examine patient-level predictors of response, AD and IPD were not combined.

#### IPD-MA

IPD were analysed in a one-stage IPD-MA using pain data at or nearest to 4 weeks of treatment (primary) [13, 14]. Secondary analyses were conducted using repeated measures data during follow-up. Model specifications for the one-stage IPD-MA were guided by assumptions made in a two-stage IPD-MA setting: model fit and computational efficiency.

The specific treatment effect was examined in a mixed effects multilevel model, clustered at the study level using a random trial intercept. Follow-up pain scores were the dependent variable. Each trial was given a separate adjustment term for baseline severity and separate residual variances. A random effects distribution was assumed for the treatment term. Potential predictors of the specific effect were examined through the addition of a stratified predictor term and a common treatment-by-predictor interaction term. For computational efficiency, the predictor, treatment and treatment-by-predictor interaction terms were assumed fixed. Two interaction terms were included: a within-study interaction term (centred to the study mean) and an across-study interaction term based on the study mean [13, 15]. The models were built using only one predictor and interaction term per model and were adjusted only for baseline pain. The parameters were estimated using the restricted maximum likelihood approach.

The model for the overall treatment effect specified separate residual variances per trial and included a random trial intercept to account for clustering at the study level. The model was developed using only the treatment arm, thus a treatment term was not used. The model was run using change-from-baseline pain scores as the dependent variable and each potential prognostic factor was entered individually as a covariate. Significant predictors (*P* < 0.05) were subsequently examined in multivariable analysis (stratified per study).

##### Secondary and sensitivity analyses

Secondary analyses were conducted using data from all durations of follow-up (repeated measures). For this, the primary models were extended to include multiple outcome data per participant by clustering also at the participant level (random intercept) and adjusting for week (fixed, common term).

A two-stage IPD-MA was conducted for sensitivity analysis and to generate forest plots using *ipdmetan* [16]. Linear regression models estimating the specific or overall treatment effects were built within each trial and subsequently pooled in a random effects MA.

All analyses were conducted in Stata software (version 15, StataCorp, College Station, TX, USA).

### Risk of bias across studies

The quality of evidence was determined using Grading of Recommendations Assessment, Development and Evaluation (GRADE) criteria [17], modified to capture quality elements relevant for IPD-MAs (Supplementary material, section GRADE modifications for IPD-MA, available at *Rheumatology* online).

## Results

### Study selection and IPD obtained

**Fig. 1:**
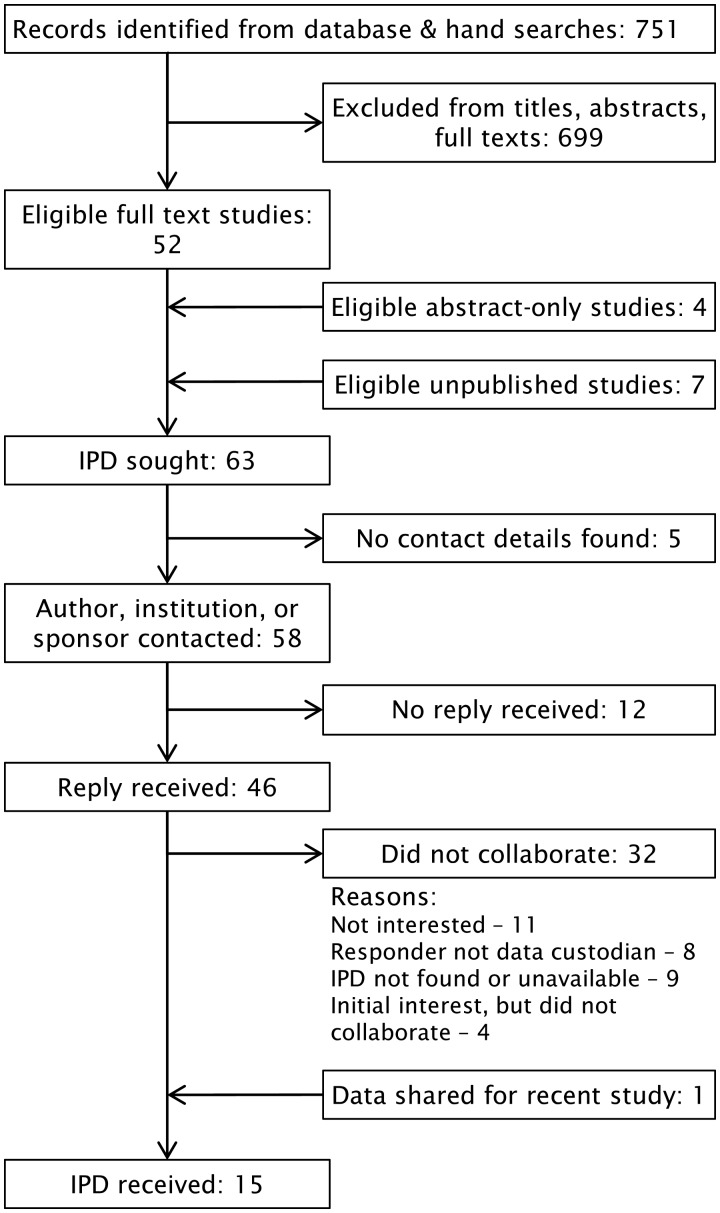
Flow chart of RCT identification, contact and acquisition for IPD-MA of topical NSAIDs in OA

IPD were sought for 63 RCTs of topical NSAIDs. Responses were obtained for 46 (Fig. 1); of these, data were received for 15 RCTs (24%). The most common reasons given for declining collaboration were not being interested (11 RCTs), not being data custodian (8 RCTs) and the IPD being unavailable or not found (9 RCTs). Direct contact with pharmaceutical manufacturers of topical NSAIDs (33 companies, including sponsors of published trials) yielded no additional data. Data collection took ∼25 months from first contact to the last data received.

Of 52 eligible RCTs available as full-text publications, 21 were analysed in the AD-MA. Reasons for exclusion were no placebo group (22 RCTs), non-eligible participants (3 RCTs) and insufficient data available in publication for analysis (6 RCTs).

Of the 15 RCTs (1951 participants on topical NSAIDs) with IPD included in this study, 11 were placebo controlled (3140 participants: 1587 on topical NSAIDs and 1553 on placebo). Active controls used were chamomile oil, SRL homeopathic gel, arnica montana gel, dwarf elder gel and any oral NSAID. Checking the received IPD confirmed that full datasets were received for 13 of the 15 RCTs, whereas 2 [18, 19] provided data only for participants who completed the trials. Across all RCTs, 6% and 5% of participants were missing primary pain data for the specific and overall analyses, respectively. Baseline demographics were balanced across participants with and without missing data, and complete case analysis was used.

### Characteristics of included studies and participants

The trial characteristics and intervention details for the 15 included RCTs were comparable to the 21 published RCTs included in the AD-MA (Supplementary Tables S2–S5, available at *Rheumatology* online). Assessments for the risk-of-bias domains are available in the Supplementary material, section Risk of bias, available at *Rheumatology* online. Randomization was adequate and fully described in 75% of the studies. This was the domain with the lowest risk of bias. Three active-controlled trials did not adequately blind participants or trial personnel.

All trials were of parallel design and recruited community-dwelling individuals (i.e. not hospital inpatients) with OA. Twelve trials were for knee OA (75% of the IPD-MA population) and three were for hand OA. The RCTs were undertaken with participants predominantly from the USA or Europe. Most (80%) trials received funding by pharmaceutical companies, including A.Vogel (BioForce AG), IBSA Institut Biochimique, Inpellis, VSM and Novartis (a GlaxoSmithKline company).

The majority of trials assessed an NSAID gel (79%), although a patch [20, 21] or cream [22] was also used. Diclofenac was the most commonly used topical NSAID, but ibuprofen and piroxicam were also assessed. One trial [23] did not specify which topical NSAID participants should use, but recommended they use ibuprofen.

Approximately two-thirds of the study population were women. The mean age was 62 years and, on average, participants reported developing OA symptoms within the preceding 3 years (Table 2).

**Table 2:** Baseline characteristics of participants

Baseline characteristic	Specific effect trials (*n* = 11)				Overall effect trials (*n* = 15)	
	*n*	NSAID	*n*	Placebo	*n*	NSAID
Randomized, *n*	1587		1553		1951	
Women, *n* (%)	1587	1077 (67.9)	1553	1085 (69.9)	1951	1324 (67.9)
Age, mean (s.d.), years	1587	62.7 (10.2)	1552	62.7 (10.4)	1951	62.5 (10.3)
Baseline pain (1–100 scale), mean (s.d.)	1586	68.0 (17.9)	1552	67.6 (18.0)	1948	65.0 (20.2)
BMI, mean (s.d.), kg/m^2^	1545	29.6 (6.2)	1516	29.7 (6.4)	1717	29.6 (6.1)
Weight, mean (s.d.), kg	1548	82.7 (19.4)	1518	82.6 (19.6)	1772	81.7 (19.1)
Inflammation (any) present, *n* (%)	1269	300 (23.6)	1238	271 (21.9)	1407	306 (21.8)
Clinical inflammation present, *n* (%)	836	152 (18.2)	825	143 (17.3)	974	158 (16.2)
Biochemical inflammation, *n* (%) per tertile	1151		1117		1141	
Lowest tertile, *n* (%)		494 (42.9)		496 (44.4)		494 (42.9)
Middle tertile, *n* (%)		325 (28.2)		300 (26.9)		325 (28.2)
Highest tertile, *n* (%)		332 (28.8)		321 (28.7)		332 (28.8)
Knee joint affected, *n* (%)	1587	1187 (74.8)	1553	1170 (75.3)	1951	1,452 (74.4)
Hand joint affected, *n* (%)	1587	400 (25.2)	1553	383 (24.7)		
Duration, median (IQR), months	152	25 (12–60)	152	27 (10–57)	136	30 (12–60)
Standardized radiographic severity, mean (s.d.)	1389	46.3 (15.2)	1360	45.4 (15.2)	184	45.4 (42.4)

Clinical signs of inflammation: presence of swelling and warmth (one study), presence of effusion (four studies). Biochemical inflammation (divided into tertiles within each study with identical observations allocated to the same tertile rank): ESR (one study), CRP (two studies). Standardized radiographic severity (0–100) calculated within each study from Kellgren–Lawrence grade (0–4; seven studies), severity of changes (0–3; one study), number of changes (1–5; one study).

Similarly, the AD-MA RCTs were all parallel design, largely recruited community-dwelling individuals from Europe or North America, were published over a similar time period, were mainly funded by pharmaceutical companies (71%) and most commonly assessed a topical NSAID gel (52%). Two-thirds of the population were women.

### Specific treatment effect

**Fig. 2:**
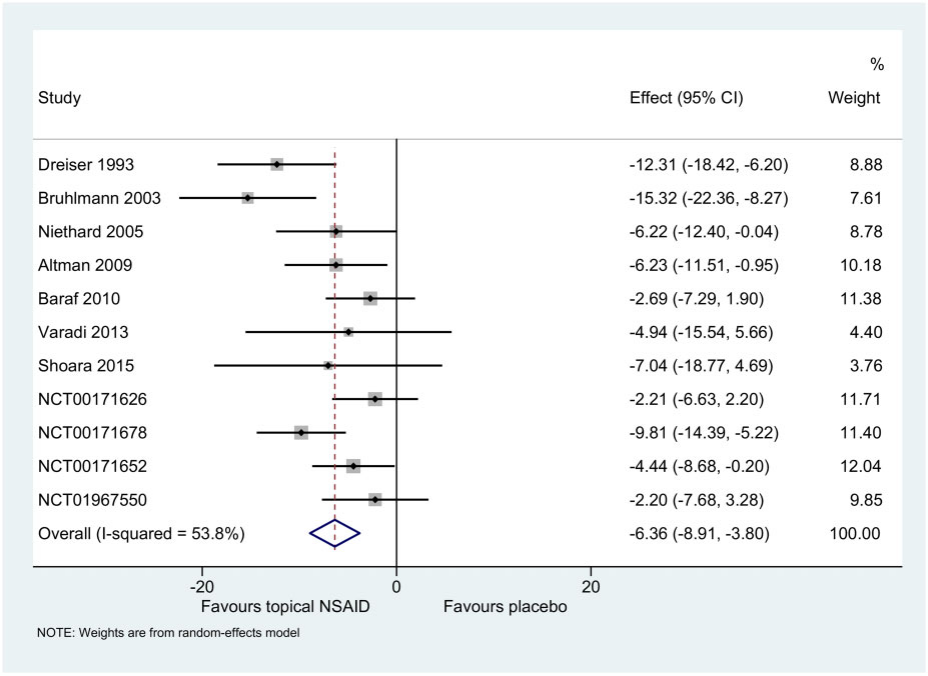
Specific treatment effect (0–100 scale) at or nearest to 4 weeks in two-stage IPD-MA. Effect presented as difference between topical NSAID and placebo on a 0–100 scale

Topical NSAIDs were statistically superior to placebo for pain relief at or nearest to 4 weeks [−6 (95% CI −9, −4)] (Fig. 2). Specific treatment effect estimates were comparable to the AD-MA [21 RCTs; 6191 participants; −8 (95% CI −10, −5); Supplementary Fig. S1, available at *Rheumatology* online]. The GRADE quality of evidence was moderate, limited by potential data availability bias (Supplementary Table S6, available at *Rheumatology* online).

### Overall treatment effect

**Fig. 3:**
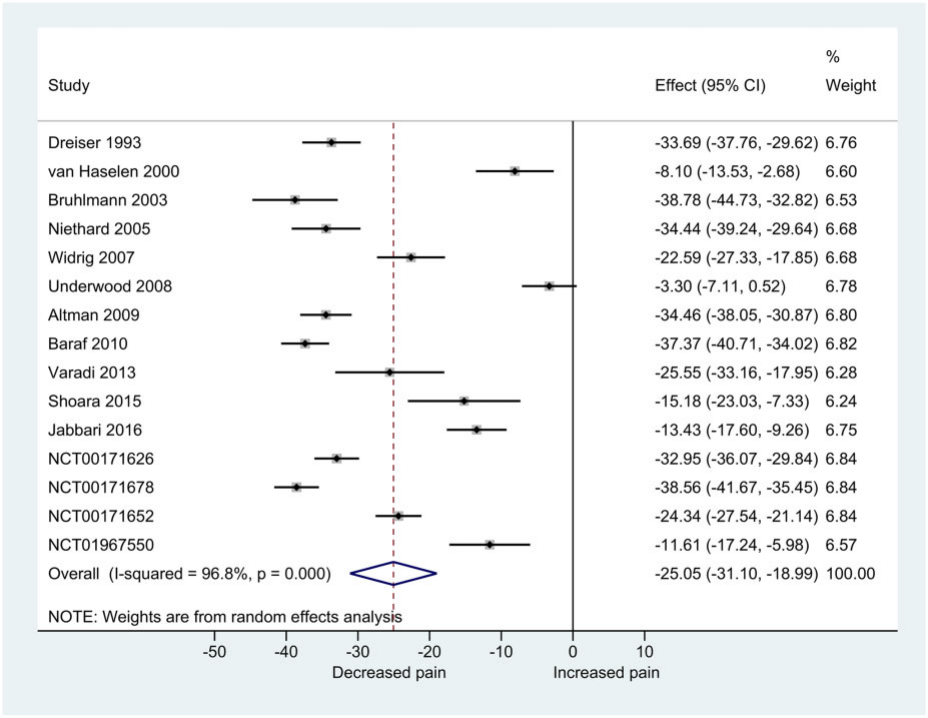
Overall treatment effect (0–100 scale) at or nearest to 4 weeks in two-stage IPD-MA. Effect presented as overall treatment effect (change from baseline) on a 0–100 scale

Participants using topical NSAIDs experienced, on average, a 25-point (95% CI −31, −19) decrease in pain at or nearest to 4 weeks (Fig. 3). A large variation in overall treatment effect was observed. The GRADE quality of evidence was deemed very low due to the study design, lack of blinding of active-controlled trials, inconsistency and potential data availability bias (Supplementary Table S7, available at *Rheumatology* online). The AD-MA estimate of the overall effect was marginally higher [21 RCTs; 3183 participants; −31 (95% CI −35, −27); Supplementary Fig. S2, available at *Rheumatology* online].

### Predictors of response

Of the pre-specified peripheral and central determinants of OA and OA pain sought from RCTs, data were only available for seven potential person-level determinants of response (Table 3). Age, BMI, inflammation, symptom duration and radiographic severity did not predict response to topical NSAIDs. A statistically significant interaction was observed between treatment and sex in the specific effect model (*P* = 0.023) (Supplementary Fig. S3, available at *Rheumatology* online), with women reporting greater reductions in pain than men [women: −7 (95% CI −10, −5) *vs* men: −3 (95% CI −6, −1)].

**Table 3:** Potential predictors of the specific and overall treatment effect in one-stage IPD-MA

Predictor	*N*	Specific effect		*N*	Overall effect	
		*n*	β (95% CI)		*n*	β (95% CI)
Sex (female = 1, male =0)	11	2939	−**4.28 (**−**7.98,** −**0.58)**	15	1857	−**3.17 (**−**5.53,** −**0.81)**
Age, years	11	2938	0.06 (−0.11, 0.22)	15	1857	−0.02 (−0.13, 0.09)
Baseline pain (0 − 100)	11	2939	0.05 (−0.06, 0.16)	15	1857	−**0.53 (**−**0.59,** −**0.47)**
BMI, kg/m^2^	10	2863	0.03 (−0.27, 0.32)	12	1633	0.09 (−0.12, 0.29)
Inflammation (yes = 1, no = 0)	7	2339	−0.31 (−4.98, 4.36)	8	1329	−2.16 (−5.42, 1.09)
Clinical	4	1550	−1.84 (−8.24, 4.55)	5	924	−3.03 (−7.40, 1.34)
Biochemical	6	2116	0.90 (−1.53, 3.33)	6	1088	1.18 (−0.55, 2.90)
Duration, months	3	280	−0.04 (−0.15, 0.07)	4	181	−0.03 (−0.09, 0.03)
XR severity (0–100)	7	2576	−0.01 (−0.12, 0.14)	9	1412	0.08 (−0.00, 0.17)

β presented on a 0–100 scale. Significant interactions (specific effect) or associations (overall effect, unadjusted model) are shown in bold. A negative interaction effect for sex (specific effect) indicates a greater specific effect for topical NSAIDs in women relative to men. A negative association between sex and overall treatment effect indicates more pain reduction in women relative to men. A negative association between baseline pain and overall treatment effect indicates that the higher the baseline pain score, the more reduction in pain.

β: beta-coefficient for interaction effect (specific effect) or covariate (overall effect); *n*: number of participants; *N*: number of trials; XR: radiographic severity.

Individuals with higher levels of pain at baseline reported significantly greater pain relief after treatment with topical NSAIDs (larger overall treatment effect, *P* < 0.001). For each 1-point increase in baseline pain, participants experienced 0.53 more pain reduction. Women also reported greater overall pain relief than men (*P* = 0.008), but adjustment for baseline pain in multivariable analysis rendered the effect of sex non-significant (*P* = 0.162). The association between baseline pain and treatment effect remained significant (*P* < 0.001).

#### Additional analyses

Participants were followed up multiple times per trial, generating 11 433 and 6494 observations for the secondary analyses of the specific and overall treatment effects, respectively. Extension of the models to include repeated measures yielded similar, but smaller, specific [−6 (95% CI −8, −4)] and overall [−20 (95% CI −27, −12)] treatment effects. Using repeated measures data in the specific effects model, the interaction between treatment and sex did not reach statistical significance [β for interaction: −3 (95% CI −6, 0); *P* = 0.066]. In the overall effects model, findings for the association between sex, baseline pain and overall treatment effects were comparable to the primary model (Supplementary Table S8, available at *Rheumatology* online).

Two-stage IPD-MAs were in agreement with findings from the one-stage models (Supplementary Tables S9 and S10, available at *Rheumatology* online).

## Discussion

To our knowledge, this is the first IPD-MA conducted to identify predictors of response to topical NSAIDs in OA. Topical NSAIDs were statistically better than placebo for OA pain (6 points on a 0–100 scale) and produced total pain reductions from baseline, including placebo effects, that were clinically significant (25 points improvement). The results were consistent across different outcome points (nearest to 4 weeks or repeated measures) and IPD-MA approaches (one-stage and two-stage IPD-MAs). Baseline pain predicted overall treatment effect, but other patient-level factors measured in RCTs of topical NSAIDs were not clinically meaningful predictors of response. Future RCTs in OA should measure additional patient characteristics that potentially may be of value for stratification of responses.

We show that participants with higher pain at baseline may report more pain relief than those with less pain at baseline. However, no interaction was observed between baseline pain and topical NSAIDs for the specific treatment effect, possibly indicating that baseline pain is a prognostic factor for the contextual or non-specific effects (such as the Hawthorn effect and regression to the mean) of topical NSAIDs [24–26]. Previous study-level evidence has shown an association between increased baseline pain severity and increased contextual responses in OA [27]. The present IPD-MA extends these findings to demonstrate that the majority of the treatment effects of topical NSAIDs derive from contextual effects and that the magnitude of contextual effects is dependent on baseline pain levels.

There is limited evidence for predictors of response to topical NSAIDs and this is the first study to present possible sex-related differences in response. The difference in specific response between the sexes was statistically significant in our primary analysis, but the effect was small and was not replicated when including data available for all measured time points. A difference in benefit between women and men of 4 points on a 0–100 point scale might not be clinically meaningful, and would not justify targeting the treatment to women alone. Effects of sex on overall treatment response might be explained by baseline pain, which was overall worse in women than men. No other measured patient-level factors significantly interacted or associated with the treatment effect of topical NSAIDs.

Additional factors not reported in the selected RCTs might better predict treatment outcomes. There is evidence that indices of central sensitization, neuropathic-like or nociceptive pain qualities, psychological factors such as catastrophizing and negative affect, radiographic OA severity or synovial inflammation associate with OA pain progression [28–31] and therefore might predict response to analgesic treatments in OA. In order to reduce research waste and facilitate future research into predictors of response, we suggest that all RCTs in OA should assess these important patient-level characteristics at baseline. These factors can be assessed by validated questionnaires, such as painDETECT (neuropathic-like pain features) [32], the Pain Catastrophizing Scale (catastrophizing) [33] and the Hospital Anxiety and Depression Scale (negative affect) [34]. Structural severity and synovial inflammation could be assessed using a combination of plain film radiographs, MRI and ultrasound. Central sensitization could be assessed using quantitative sensory testing. Standardizing the measurement of a core set of potentially important factors across RCTs would ensure that future IPD-MAs are able to examine factors that are of theoretical importance to identifying predictors of response to analgesics.

We suggest that patients with knee and hand OA may benefit from trying a topical NSAID, as overall treatment effects are large and pain reduction is likely to be clinically significant. Topical NSAIDs may be considered in a patient with OA, irrespective of their age, BMI, level of inflammation, duration of complaints and radiographic severity. Patients with more severe pain at baseline may experience larger overall levels of pain relief and thus warrant trying a topical NSAID before moving up the analgesic ladder. Finally, the difference in effect between men and women is unlikely to be clinically significant, and topical NSAIDs should continue to be offered equally irrespective of gender.

The present IPD-MA is subject to several limitations. Only a subset of eligible studies were analysed and the present work may therefore be subject to data availability bias, reflected in the GRADE rating of quality [35]. However, the specific effect IPD-MA still included a large pool of participants (*n* = 3140), and participant characteristics and outcomes were similar in our IPD to our AD-MA, suggesting that the included studies might be representative of the eligible study pool. Although the participant characteristics analysed were defined *a priori*, multiple covariates were examined and our examination of predictors should be viewed as exploratory. In order to pool data, pain outcome scores were standardized from their original scales to a 0–100 scale, as in previous studies [10, 36], although the instruments might have different measurement properties or sensitivities. Data quality was not high, although quality downgrading was partly due to analyses of the overall treatment within one treatment arm, thereby making the data observational in nature. Our model specifications were guided by assumptions made in the two-stage IPD-MA [14], and this might have influenced the results. Due to model complexity, assumptions were further limited by non-convergence, and intention-to-treat analysis could not be conducted because of model complexity.

In conclusion, topical NSAIDs are effective for OA pain. People with higher OA pain at baseline experience greater overall reductions in pain on using the treatment, but this may be attributed to contextual or non-specific, rather than specific, treatment effects. Other baseline characteristics routinely reported in RCTs did not predict clinically important differences in topical NSAID response. Additional factors that have been linked either mechanistically or through empirical evidence to outcomes should be selected for inclusion across future RCTs in order to facilitate the identification of response predictors through IPD-MA. Such factors might include recognized central and peripheral risk factors for OA pain.

**Rheumatology key messages**

- Topical NSAIDs are effective for OA pain.
- No clinically significant predictors of the specific treatment response are available in published RCTs.
- Future RCTs should measure recognized peripheral/central risk factors for OA pain at baseline.

## Supplementary Material

keaa113_Supplementary_Data
