# Supplementary material for: Predicting response to topical non-steroidal anti-inflammatory drugs in osteoarthritis: an individual patient data meta-analysis of randomized controlled trials
Source: Rheumatology (Oxford). 2020 Apr 10;59(9):2207–16. doi: 10.1093/rheumatology/keaa113 (PMC7449808; doi:10.1093/rheumatology/keaa113)
Supplement: keaa113_Supplementary_Data [file keaa113_supplementary_data.zip › keaa113-Suppl_Data/rhe-19-1496-File008.docx]

**SUPPLEMENTARY MATERIAL**

## Example literature search strategy

| **Topical NSAIDs search strategy: Medline (via Ovid)** |
| --- |
| 1. Randomized Controlled Trials as Topic/  2. (randomi?ed controlled trial or double-blind or blind$ or mask$ or clinical trial or trial).af.  3. Administration, Topical/  4. (stick-on or cutaneous or dermal or transcutaneous or percutaneous or skin or massage or embrocation or gel or ointment or aerosol or cream or lotion or mousse or foam or liniment or spray or rub or balm or salve or emulsion or oil or patch or plaster).af.  5. exp Osteoarthritis/dt, th [Drug Therapy, Therapy]  6. (osteoarthr* or OA or arthritis or Degenerative arthritis or degenerative joint disease or arthrosis or osteoarthrosis).af.  7. Anti-Inflammatory Agents, Non-Steroidal/ad, ae, tu [Administration & Dosage, Adverse Effects, Therapeutic Use]  8. (nsaid or nonsteroidal antiinflammatory or non-steroidal anti-inflammatory or bufexamac or bufexine or calmaderm or ekzemase or dicoflenac or solaraze or pennsaid or voltarol or emulgen or voltarene or optha or voltaren or etofenamate or afrolate or algesalona or bayro or deiron or etofen or flexium or flogoprofen or rheuma-gel or rheumon or traumalix or traumon or zenavan or felbinac or dolinac or flexfree or napageln or target or traxam or fentiazac or domureuma or fentiazaco or norvedan or riscalon or fepradinol or dalgen or flexidol or cocresol or rangozona or reuflodol or pinazone or zepelin or flufenamic or dignodolin or rheuma or lindofluid or sastridex or lunoxaprofen or priaxim or flubiprofen or fenomel or ocufen or ocuflur or "trans act lat" or tulip or ibuprofen or cuprofen or "deep relief" or fenbid or ibu-cream or ibugel or ibuleve or ibumousse or ibuspray or "nurofen gel" or proflex or motrin or advil or radian or ralgex or ibutop or indomethacin or indocin or indospray or isonixin or nixyn or ketoprofen or tiloket or oruvail or powergel or solpaflex or ketorolac or acular or trometamol or meclofenamic or naproxen or naprosyn or niflumic or actol or flunir or niflactol topico or niflugel or nifluril or oxyphenbutazone or californit or diflamil or otone or tanderil or piketoprofen or calmatel or triparsean or piroxicam or feldene or pranoprofen or oftalar or pranox or suxibuzone or danilon or flamilon or ufenamate or fenazol).af.  9. 1 or 2  10. 3 or 4  11. 5 or 6  12. 7 or 8  13. 9 and 10 and 11 and 12  14. Limit 13 to randomized controlled trial |
|  |

## Supplementary Table S1. Methods for standardising and pooling variables for IPD MA

| Variable | Notes for standardisation |
| --- | --- |
| Sex | Female/male |
| Age | Years |
| Baseline pain | 0-100 standardised scale per study |
| BMI | kg/m^2^ |
| Duration of complaints | Months |
| Clinical inflammation | Clinical signs of inflammation were recorded the presence or absence of clinical signs of inflammation in the form of effusion (NCT00171626, (1-3)) or warmth and swelling(4) of the affected joint. |
| Biochemical inflammation | Biochemical markers of inflammation used were erythrocyte sedimentation rate (ESR)(5) or C-reactive protein (CRP)(NCT00171652, NCT00171626,(1, 2, 6)). Although insensitive, these markers were used as surrogates for inflammation and were divided into tertiles per trial (highest to lowest level of blood marker) before pooling. Identical observations were allocated the same tertile rank |
| Overall marker of inflammation | In order to generate an overall indicator of inflammation, clinical and blood markers were combined. The blood markers were dichotomised using the person-specific thresholds of normality specified within the primary trials. |
| Radiographic severity | Radiographic OA severity was assessed in nine trials using different criteria. Seven studies presented KL grades (NCT00171652, NCT00171626, NCT01967550, (1, 2, 6, 7)), one study rated the severity of changes,(8) and the last study reported only the number of changes.(3) In order to pool the radiographic severity grades across trials, these were first standardised to a 0-100 scale per trial. |
| Others | No trials assessed the quality of pain or presence of neuropathic-like pain. Only one trial(4) recorded the presence of pain elsewhere (a possible indicator for central mechanisms of pain) and this was therefore not analysed. |

## GRADE modifications for IPD MA

**Study design**

Examination of the specific effect was conducted using randomised comparisons, so initial quality commenced as high. For the overall treatment effect, only one treatment arm is analysed. The data are therefore rendered observational as no randomised comparison remains. The initial quality of the evidence was therefore low.

**Risk of bias**

Traditional risk of bias domains from published manuscripts do not translate directly to IPD MAs. Risks of bias were assessed using Cochrane’s framework, as described below:

- Selection, performance, and detection bias were determined as specified by Cochrane
- Incomplete accounting of participants was not based on how missing outcome data were handled in the original trial publications (e.g., if ITT was used). As data were re-analysed in the IPD MA, the quantity of missing outcome data were examined. The characteristics of those with and without missing data were compared and the subsequent risk of bias was judged from this
- Rather than assessing selective outcome reporting, assessment addressed whether trialists withheld data on variables/outcomes that were requested and known to have been measured in the trial in question

**Inconsistency**

One-stage IPD MAs do not readily generate output comparable to AD MAs with regards to tests of heterogeneity and I^2^. Inconsistency was therefore ascertained from visual examination of the forest plots

**Indirectness**

Appraised as per GRADE guidelines. Note that in the IPD MA setting, inclusion/exclusion criteria can be applied at the individual level to make the population tested similar to the population of interest.

**Imprecision**

Appraised as per GRADE guidelines.

**Publication bias**

Rather than considering publication bias alone, data availability bias was considered. Data retrieval rates were calculated and the results of the IPD MA were compared to the published database (AD MA). Where a significant proportion of IPD were not received, important differences were observed between trials accepting or declining collaboration, or the findings from the IPD MA differed significantly from the AD MA, the evidence was downgraded for potential concerns of data availability bias.

## Supplementary Table S2. Descriptions of trials analysed in IPD MA

| Trial Name | Year | Country of Study | Funding source | Design | Setting | Joints Affected | Week analysed |
| --- | --- | --- | --- | --- | --- | --- | --- |
| Placebo-controlled trials | | | | | | |  |
| Dreiser 1993 | 1993 | France | Commercial | Parallel | Community | Knee | 2 |
| Bruhlmann 2003 | 2003 | Switzerland | Commercial | Parallel | Community | Knee | 2 |
| Niethard 2005 | 2005 | Germany | Commercial | Parallel | Community | Knee | 3 |
| Altman 2009 | 2009 | USA | Commercial | Parallel | Community | Hand | 4 |
| Baraf 2010 | 2010 | USA | Commercial | Parallel | Community | Knee | 4 |
| Varadi 2013 | 2013 | Switzerland | Commercial | Parallel | Community | Knee | 2 |
| Shoara 2015 | 2015 | Iran | Public | Parallel | Community | Knee | 3 |
| NCT00171626 | 2005* | USA | Commercial | Parallel | Community | Knee | 4 |
| NCT00171678 | 2005* | USA | Commercial | Parallel | Community | Knee | 4 |
| NCT00171652 | 2005* | Germany, France | Commercial | Parallel | Community | Hand | 4 |
| NCT01967550 | 2014* | Germany | Commercial | Parallel | Community | Knee | 4 |
| Active-controlled trials | | | | | | |  |
| Van Haselen 2000 | 2000 | UK | Commercial | Parallel | Community | Knee | 4 |
| Widrig 2007 | 2007 | Switzerland | Commercial | Parallel | Community | Hand | 3 |
| Underwood 2008 | 2008 | UK | Public | Parallel | Community | Knee | 13 |
| Jabbari 2016 | 2016 | Iran | Public | Parallel | Community | Knee | 4 |
| Year is year of publication unless denoted by “*”. Those with “*” were not published as a stand-alone RCT publication, so year is date of trial.  Week analysed is the time point analysed for the primary analysis at or nearest to four weeks.  Data from NCT00171652 was included in the publication by Barthel, Peniston (9) which pooled two RCTs, whilst NCT00171626 and NCT00171678 were pooled in the publication by Baraf, Gloth (10). NCT01967550 remains unpublished | | | | | | | |

| Trial Name | Intervention | Strength | Formulation | Frequency per Day | Comparator(s) |
| --- | --- | --- | --- | --- | --- |
| Placebo-controlled trials | | | | | |
| Dreiser 1993 | Diclofenac hydroxyethylpyrrolidine | 180mg | Patch | 2 | Placebo |
| Bruhlmann 2003 | Diclofenac epolamine | 180mg | Patch | 2 | Placebo |
| Niethard 2005 | Diclofenac diethylamine | 1.16% | Gel | 4 | Placebo |
| Altman 2009 | Diclofenac sodium | 1% | Gel | 4 | Placebo |
| Baraf 2010 | Diclofenac sodium | 1% | Gel | 4 | Placebo |
| Varadi 2013 | Ibuprofen-VALE | 10% | Cream | 2 | Placebo |
| Shoara 2015 | Diclofenac sodium | 1% | Gel | 3 | Placebo, chamomile oil |
| NCT00171626 | Diclofenac sodium | 1% | Gel | 4 | Placebo |
| NCT00171678 | Diclofenac sodium | 1% | Gel | 4 | Placebo |
| NCT00171652 | Diclofenac sodium | 1% | Gel | 4 | Placebo |
| NCT01967550 | Diclofenac diethylamine | 2.32% | Gel | 2 | Placebo |
| Active-controlled trials | | | | | |
| Van Haselen 2000 | Piroxicam | 0.5% | Gel | 3 | SRL homeopathic gel |
| Widrig 2007 | Ibuprofen | 5% | Gel | 3 | Arnica montana gel |
| Underwood 2008 | Any topical NSAID | - | - | - | Any oral NSAID |
| Jabbari 2016 | Diclofenac sodium | 1% | Gel | 3 | Dwarf elder gel |
| NSAID, non-steroidal anti-inflammatory drug | | | | | |

## Supplementary Table S3. Intervention details for the trials analysed in the IPD MA

## Supplementary Table S4. Trial details of the published RCTs included in the AD MA

| Trial Name | Year | Country of Study | Funding source | Design | Setting | Joints Affected |
| --- | --- | --- | --- | --- | --- | --- |
| Dreiser 1993 | 1993 | France | Unclear | Parallel | Community | Knee |
| Yoo 1996 | 1996 | Korea | Unclear | Parallel | Community | Knee |
| Sandelin 1997 | 1997 | Finland, Sweden | Unclear | Parallel | Community | Knee |
| Grace 1999 | 1999 | Canada | Public, Commercial | Parallel | Unclear | Knee |
| Rovensky 2001 | 2001 | Slovakia | Unclear | Parallel | Community | Knee |
| Ottillinger 2001 | 2001 | Hungary, Czech Republic, Switzerland, Germany | Commercial | Parallel | Community | Knee |
| Bruhlmann 2003 | 2003 | Switzerland | Unclear | Parallel | Community | Knee |
| Roth 2004 | 2004 | USA | Commercial | Parallel | Community | Knee |
| Trnavsky 2004 | 2004 | Czech Republic | Commercial | Parallel | Community | Knee |
| Bookman 2004 | 2004 | Canada | Commercial | Parallel | Community | Knee |
| Baer 2005 | 2005 | Canada | Commercial | Parallel | Community | Knee |
| Niethard 2005 | 2005 | Germany | Commercial | Parallel | Community | Knee |
| Rother 2007 | 2007 | Germany | Commercial | Parallel | Community | Knee |
| Simon 2009 | 2009 | Canada, USA | Commercial | Parallel | Community | Knee |
| Altman 2009 | 2009 | USA | Commercial | Parallel | Unclear | Hand |
| Baraf 2010 | 2010 | USA | Commercial | Parallel | Community | Knee |
| Kneer 2013 | 2013 | Germany, Poland, Serbia, Croatia | Commercial | Parallel | Community | Knee |
| Rother 2013 | 2013 | USA | Commercial | Parallel | Community | Knee |
| Varadi 2013 | 2013 | Switzerland | Commercial | Parallel | Community | Knee |
| Conaghan 2013 | 2013 | Czech Republic, Germany, Poland, UK | Commercial | Parallel | Community | Knee |
| Shoara 2015 | 2015 | Iran | Public | Parallel | Community | Knee |
| Year is year of publication | | | | | | |

## Supplementary Table S5. Intervention details for the published RCTs included in the AD MA

| Trial Name | Intervention (Carrier) | Strength | Formulation | Frequency per Day | Comparator(s) |
| --- | --- | --- | --- | --- | --- |
| Dreiser 1993 | Diclofenac hydroxyethylpyrrolidine | 180mg | Patch | 2 | Placebo |
| Yoo 1996 | Ketoprofen |  | Patch | 2 | Placebo |
| Sandelin 1997 | Eltenac | 1% | Gel | 3 | Placebo (oral + gel), oral diclofenac |
| Grace 1999 | Diclofenac sodium (pluronic lecithin) | 2% | Gel | 3 | Placebo (pluronic lecithin vehicle) |
| Rovensky 2001 | Ibuprofen | 5% | Cream | 3 | Placebo |
| Ottillinger 2001 | Eltenac | 1%, 0.3%, 0.1% | Gel | 3 | Placebo |
| Bruhlmann 2003 | Diclofenac epolamine | 180mg | Patch | 2 | Placebo |
| Roth 2004 | Diclofenac sodium (DMSO) | 1.5% | Solution | 4 | Placebo (45.5% DMSO) |
| Bookman 2004 | Diclofenac sodium (DMSO) | 1.5% | Solution | 4 | Placebo (4.55% DMSO), placebo (45.5% DMSO) |
| Trnavsky 2004 | Ibuprofen | 5% | Cream | 3 | Placebo |
| Niethard 2005 | Diclofenac diethylamine | 1.16% | Gel | 4 | Placebo |
| Baer 2005 | Diclofenac sodium (DMSO) | 1.5% | Solution | 4 | Placebo (45.5% DMSO) |
| Rother 2007 | Ketoprofen (Transfersome) | 110mg | Gel | 2 | Placebo (gel + oral), oral celecoxib |
| Altman 2009 | Diclofenac sodium | 1% | Gel | 4 | Placebo |
| Simon 2009 | Diclofenac sodium (DMSO) | 1.5% | Solution | 4 | Placebo (oral + modified 2.3% DMSO)**, placebo (45.5% DMSO)**, oral diclofenac**, oral diclofenac + topical diclofenac sodium |
| Baraf 2010 | Diclofenac sodium | 1% | Gel | 4 | Placebo |
| Continued overleaf | | | | | |
| Trial Name | **Intervention (Carrier)** | **Strength** | **Formulation** | **Frequency per Day** | **Comparator(s)** |
| Rother 2013 | Ketoprofen (Transfersome) | 100mg | Gel | 2 | Placebo (4.4 g Transfersome Vehicle) |
| Conaghan 2013 | Ketoprofen (Transfersome) | 50mg, 100mg | Gel | 2 | Placebo (oral), placebo (2.2 g Transfersome), placebo (4.4 g Transfersome), oral celecoxib |
| Varadi 2013 | Ibuprofen-VALE | 10% | Cream | 2 | Placebo |
| Kneer 2013 | Ketoprofen | 25mg, 50mg, 100mg | Gel | 2 | Placebo |
| Shoara 2015 | Diclofenac sodium | 1% | Gel | 3 | Placebo, chamomile oil |
| DMSO, dimethylsulfoxide | | | | | |

## Risk of bias

Risk of bias assessments were conducted for the 15 included trials using information available in the published reports (**Figure 1**). Three trials were published as post hoc analyses of multiple RCTs conducted by the same pharmaceutical sponsor using similar methods. These reports(9, 10) were used for the risk of bias assessment. One trial (NCT01967550) has not been published and risk of bias assessment was deemed “unclear” across all domains.

Random number sequence generation was fully described and deemed adequate in 12 of 15 trials. This was the domain associated with the lowest risk of bias. However, randomisation did not successfully balance the proportion of women in two RCTs(7, 11) or WOMAC scores in one RCT(5). Three active-controlled trials did not adequately blind participants and/or trial personnel.(4, 8, 11)

The domain with the highest risk of bias was the use of ITT. Approximately two-thirds of trials did not analyse all randomised participants in the published reports.

| Supplementary Table S6. GRADE assessment for specific effect of topical NSAIDs | | | |
| --- | --- | --- | --- |
| GRADE criteria | Rating | Footnotes | Quality of evidence |
| Study design | High | RCTs | ⊕⊕⊕⊙ Moderate |
| Risk of Bias | No | **Randomisation and treatment allocation** – likely low risk of bias across trials. Analysis adjusts for any residual differences in baseline pain severity. **Blinding** – all but one trial (n=56) were adequately blinded. Less than 2% of the population was unblinded, so unlikely to substantially bias results. **Selective outcome reporting** – all trials contributed data and baseline variables as requested. **Incomplete accounting of patients** – ITT not used, but overall withdrawal rates were low. No differences were observed in baseline characteristics between drop-outs and those that completed |  |
| Inconsistency | No | There is some inconsistency. The ES vary from -15.32 to -2.20 in the included studies, but the majority of 95%CIs overlap. The inconsistency is unlikely to impact decision making, so evidence quality is not downgraded. The inconsistency may be explained by differences between formulations (patch ES -13.60, 95%CI -18.22 to -8.99 versus cream/gel ES -4.84, 95%CI -6.88 to -2.80), but the aim of the work is to examine topical NSAIDs overall, not by formulation. The results are therefore not presented by subgroup. Note that I^2^ is not generated in one-stage IPD MA and was not used. |  |
| Indirectness | No | PICO match MA aims. |  |
| Imprecision | No | Overall sample size is 3,140, this is likely sufficient. All of 95%CI is held within the same range of interpretation (statistically significant, but not clinically significant) |  |
| Publication/ data availability bias | Serious (-1) | Data only received for approximately 35% of placebo-controlled trials. There is a range of small and large studies, including unpublished data. Comparison of AD MA results between trials that shared IPD (Hedges’ ES 0.42, 95%CI 0.23 to 0.61) and those that didn’t (Hedges’ ES 0.25, 95%CI 0.13 to 0.38), indicated there may be some data availability bias. However, IPD were received from trials not included in the AD MA, limiting the comparison between the IPD MA and AD MA to determine data availability bias. However, both provided similar specific effect estimates. The evidence is downgraded primarily because of the poor data retrieval rate. The IPD may be reflective of the overall evidence, but we cannot confidently determine this. |  |

## Supplementary Table S7. GRADE assessment for overall treatment effect of topical NSAIDs

| GRADE criteria | Rating | Footnotes | Quality of evidence |
| --- | --- | --- | --- |
| Study design | Low | Observational | ⊕⊙⊙⊙ Very low |
| Risk of Bias | Serious (-1) | Randomisation and treatment allocation – likely low risk of bias across trials.  Blinding – three trials (n=258) did not adequately blind participants as they compared topical NSAIDs to an active comparator using a different formulation. With 13% of the population not blinded it may have biased the findings. Especially as the unblinded trials generally reported lower overall treatment effects  Selective outcome reporting – not relevant for IPD MA as all trials contributed pain data  Incomplete accounting of patients – ITT not used, but overall withdrawal rates were low. No differences were observed in baseline characteristics between drop-outs and those that completed |  |
| Inconsistency | Serious (-1) | Inconsistency is observed. ES estimates vary from -38.78 to -3.30. Most 95%CI do not overlap. However, Note that I^2^ is not generated in one-stage IPD MA and was not used. |  |
| Indirectness | No | PICO match MA aims. |  |
| Imprecision | No | Overall sample size is 1,951, this is likely sufficient  All of 95%CI is held within the same range of interpretation (statistically and clinically significant) |  |
| Publication/data availability bias | Serious (-1) | Data received for 24% of topical NSAID trials (irrespective of type of comparator). A mixture of small and large trials, including some unpublished data, were included. Estimate is conservative, but similar to overall effect from ADMA (using placebo-controlled trials). However, due to the poor data retrieval rates, the evidence is downgraded |  |

## Supplementary Table S8. Predictors of the specific and overall treatment effect at all time points (repeated measures)

|  | Specific effect | | | | |  | Overall effect | | | | |
| --- | --- | --- | --- | --- | --- | --- | --- | --- | --- | --- | --- |
| Predictor | **N** | **n** | **Obs** | **ß** | **95%CI** |  | **N** | **n** | **Obs** | **ß** | **95%CI** |
| Sex | 11 | 3,097 | 11,433 | -2.94 | -6.07 to 0.20 |  | 15 | 1,925 | 6,494 | **-2.93** | **-5.12 to -0.75** |
| Age | 11 | 3,096 | 11,431 | 0.07 | -0.08 to 0.21 |  | 15 | 1,925 | 6,494 | 0.00 | -0.10 to 0.10 |
| Baseline pain | 11 | 3,097 | 11,433 | 0.01 | -0.08 to 0.10 |  | 15 | 1,925 | 6,494 | **-0.55** | **-0.61 to -0.50** |
| BMI | 10 | 3,019 | 11,195 | 0.07 | -0.18 to 0.31 |  | 12 | 1,699 | 6,183 | 0.10 | -0.08 to 0.28 |
| Inflammation | 7 | 2,477 | 9,524 | -0.67 | -4.57 to 3.23 |  | 8 | 1,391 | 5,273 | -1.66 | -4.42 to 1.09 |
| Clinical | 4 | 1,633 | 5,759 | -2.50 | -7.86 to 2.87 |  | 5 | 959 | 3,343 | -3.04 | -6.77 to 0.69 |
| Biochemical | 6 | 2,247 | 8,843 | 1.01 | -1.04 to 3.06 |  | 6 | 1,143 | 4,537 | 0.65 | -0.83 to 2.12 |
| Duration | 3 | 292 | 626 | -0.00 | -0.09 to 0.09 |  | 4 | 182 | 389 | -0.01 | -0.07 to 0.05 |
| XR severity | 7 | 2,719 | 10,637 | -0.02 | -0.12 to 0.09 |  | 9 | 1,473 | 5,565 | 0.04 | -0.04 to 0.12 |
| Significant interactions (specific effect) or associations (overall effect, univariate analysis) are shown in bold.  ß, beta-coefficient for interaction effect (specific effect) or covariate (overall effect) presented on 0-100 scale; BMI, body mass index; CI, confidence interval; n, number of participants; N, number of trials; Obs, number of observations; XR, radiographic severity | | | | | | | | | | | |

## Supplementary Table S9. Specific treatment effect and its predictors in one-stage versus two-stage IPD MA

|  | One-stage | | Two-stage | |
| --- | --- | --- | --- | --- |
| Examination | **ß** | **95%CI** | **ß** | **95%CI** |
| At or nearest to four weeks | **-6.35** | **-8.89 to -3.81** | **-6.36** | **-8.91 to -3.80** |
| Baseline interactions |  |  |  |  |
| Sex | **-4.28** | **-7.98 to -0.58** | **-4.23** | **-7.88 to -0.58** |
| Age | 0.06 | -0.11 to 0.22 | 0.05 | -0.11 to 0.22 |
| Baseline pain | 0.05 | -0.06 to 0.16 | 0.04 | -0.06 to 0.15 |
| BMI | 0.03 | -0.27 to 0.32 | 0.01 | -0.29 to 0.31 |
| Inflammation | -0.31 | -4.98 to 4.36 | -0.46 | -5.09 to 4.17 |
| Clinical | -1.84 | -8.24 to 4.55 | -2.47 | -10.32 to 5.39 |
| Biochemical | 0.90 | -1.53 to 3.33 | 1.07 | -2.47 to 4.61 |
| Duration | -0.04 | -0.15 to 0.07 | -0.05 | -0.19 to 0.09 |
| XR severity | -0.01 | -0.12 to 0.14 | 0.01 | -0.14 to 0.16 |
|  |  |  |  |  |
| Repeated measures | **-6.19** | **-8.36 to -4.03** | **-7.02** | **-9.97 to -4.06** |
| Baseline interactions |  |  |  |  |
| Sex | -2.94 | -6.07 to 0.20 | -2.68 | -5.68 to 0.33 |
| Age | 0.07 | -0.08 to 0.21 | 0.04 | -0.10 to 0.18 |
| Baseline pain | 0.01 | -0.08 to 0.10 | 0.00 | -0.09 to 0.09 |
| BMI | 0.07 | -0.18 to 0.31 | 0.06 | -0.22 to 0.33 |
| Inflammation | -0.67 | -4.57 to 3.23 | -0.68 | -4.41 to 3.04 |
| Clinical | -2.50 | -7.86 to 2.87 | -2.48 | -7.61 to 2.66 |
| Biochemical | 1.01 | -1.04 to 3.06 | 1.28 | -1.27 to 3.82 |
| Duration | -0.00 | -0.09 to 0.09 | -0.00 | -0.09 to 0.09 |
| XR severity | -0.02 | -0.12 to 0.09 | -0.03 | -0.17 to 0.12 |

## Supplementary Table S10. Overall treatment effect and its predictors in one-stage versus two-stage IPD MA

|  | One-stage | | Two-stage | |
| --- | --- | --- | --- | --- |
| Examination | **ß** | **95%CI** | **ß** | **95%CI** |
| At or nearest to four weeks | **-25.04** | **-31.14 to -18.95** | **-25.05** | **-31.10 to -18.99** |
| Baseline associations |  |  |  |  |
| Sex | **-3.17** | **-5.53 to -0.81** | **-3.20** | **-5.57 to -0.83** |
| Age | -0.02 | -0.13 to 0.09 | -0.01 | -0.12 to 0.10 |
| Baseline pain | **-0.53** | **-0.59 to -0.47** | **-0.50** | **-0.63 to -0.37** |
| BMI | 0.09 | -0.12 to 0.29 | 0.12 | -0.23 to 0.46 |
| Inflammation | -2.16 | -5.42 to 1.09 | -2.23 | -5.87 to 1.42 |
| Clinical | -3.03 | -7.40 to 1.34 | -3.42 | -9.48 to 2.64 |
| Biochemical | 1.18 | -0.55 to 2.90 | 1.24 | 0.98 to -3.45 |
| Duration | -0.03 | -0.09 to 0.03 | -0.03 | -0.10 to 0.03 |
| XR severity | 0.08 | -0.00 to 0.17 | 0.08 | -0.03 to 0.18 |
| Repeated measures | **-19.44** | **-27.03 to -11.86** | - | - |
| Baseline associations |  |  |  |  |
| Sex | **-2.93** | **-5.12 to -0.75** | **-2.82** | **-4.92 to -0.73** |
| Age | 0.00 | -0.10 to 0.10 | -0.02 | -0.13 to 0.10 |
| Baseline pain | **-0.55** | **-0.61 to -0.50** | **-0.48** | **-0.60 to -0.36** |
| BMI | 0.10 | -0.08 to 0.28 | 0.12 | -0.14 to 0.38 |
| Inflammation | -1.66 | -4.42 to 1.09 | -1.82 | -4.74 to 1.09 |
| Clinical | -3.04 | -6.77 to 0.69 | -3.20 | -6.87 to 0.48 |
| Biochemical | 0.65 | -0.83 to 2.12 | 0.86 | -1.06 to 2.79 |
| Duration | -0.01 | -0.07 to 0.05 | -0.04 | -0.10 to 0.02 |
| XR severity | 0.04 | -0.04 to 0.12 | 0.05 | -0.05 to 0.15 |

**Supplementary figure legend**

**Figure 1 – Risk of bias assessment of included trials**

Figure S1. AD MA for topical NSAIDs versus placebo for pain relief at or nearest to four weeks.

ES presented as standardised mean difference (SMD) calculated using Hedges’ correction.

## Figure S2. AD MA for the overall treatment effect (Hedges’ ES) of topical NSAIDs at or nearest to four weeks in published placebo-controlled trials

Figure S3 - Topical NSAID-by-sex interaction effect in two-stage IPD MA. Interaction effect presented on 0-100 scale. A negative interaction effect represents greater efficacy in women compared to men

## Reference list

1. Barthel HR, Haselwood D, Longley S, Gold MS, Altman RD. Randomized controlled trial of diclofenac sodium gel in knee osteoarthritis. Seminars in arthritis and rheumatism. 2009;39(3):203-12.

2. Baraf HSB, Gold MS, Clark MB, Altman RD. Safety and Efficacy of Topical Diclofenac Sodium 1% Gel in Knee Osteoarthritis: A Randomized Controlled Trial. Physician and Sportsmedicine. 2010;38(2):19-28.

3. Niethard FU, Gold MS, Solomon GS, Liu JM, Unkauf M, Albrecht HH, et al. Efficacy of topical diclofenac diethylamine gel in osteoarthritis of the knee. Journal of Rheumatology. 2005;32(12):2384-92.

4. Underwood M, Ashby D, Cross P, Hennessy E, Letley L, Martin J, et al. Advice to use topical or oral ibuprofen for chronic knee pain in older people: randomised controlled trial and patient preference study. BMJ. 2008;336(7636):138-42.

5. Varadi G, Zhu Z, Blattler T, Hosle M, Loher A, Pokorny R, et al. Randomized clinical trial evaluating transdermal Ibuprofen for moderate to severe knee osteoarthritis. Pain Physician. 2013;16(6):749-62.

6. Altman RD, Dreiser RL, Fisher CL, Chase WF, Dreher DS, Zacher J. Diclofenac Sodium Gel in Patients with Primary Hand Osteoarthritis: A Randomized, Double-blind, Placebo-controlled Trial. Journal of Rheumatology. 2009;36(9):1991-9.

7. Jabbari M, Hashempur M, Razavi S, Shahraki H, Kamalinejad M, Emtiazy M. Efficacy and short-term safety of topical Dwarf Elder (Sambucus ebulus L.) versus diclofenac for knee osteoarthritis: a randomized, double-blind, active-controlled trial. Journal of Ethnopharmacology. 2016;188:80-6.

8. van Haselen RA, Fisher PA. A randomized controlled trial comparing topical piroxicam gel with a homeopathic gel in osteoarthritis of the knee. Rheumatology. 2000;39(7):714-9.

9. Barthel HR, Peniston JH, Clark MB, Gold MS, Altman RD. Correlation of pain relief with physical function in hand osteoarthritis: randomized controlled trial post hoc analysis. Arthritis Research & Therapy. 2010;12(1):R7.

10. Baraf HSB, Gloth FM, Barthel HR, Gold MS, Altman RD. Safety and efficacy of topical diclofenac sodium gel for knee osteoarthritis in elderly and younger patients: Pooled data from three randomized, double-blind, parallel-group, placebo-controlled, multicentre trials. Drugs and Aging. 2011;28(1):27-40.

11. Shoara R, Hashempur MH, Ashraf A, Salehi A, Dehshahri S, Habibagahi Z. Efficacy and safety of topical Matricaria chamomilla L.(chamomile) oil for knee osteoarthritis: a randomized controlled clinical trial. Complementary Therapies in Clinical Practice. 2015;21(3):181-7.
