# Supplementary figures and images for: Predicting response to topical non-steroidal anti-inflammatory drugs in osteoarthritis: an individual patient data meta-analysis of randomized controlled trials
Source: Rheumatology (Oxford). 2020 Apr 10;59(9):2207–16. doi: 10.1093/rheumatology/keaa113 (PMC7449808; doi:10.1093/rheumatology/keaa113)

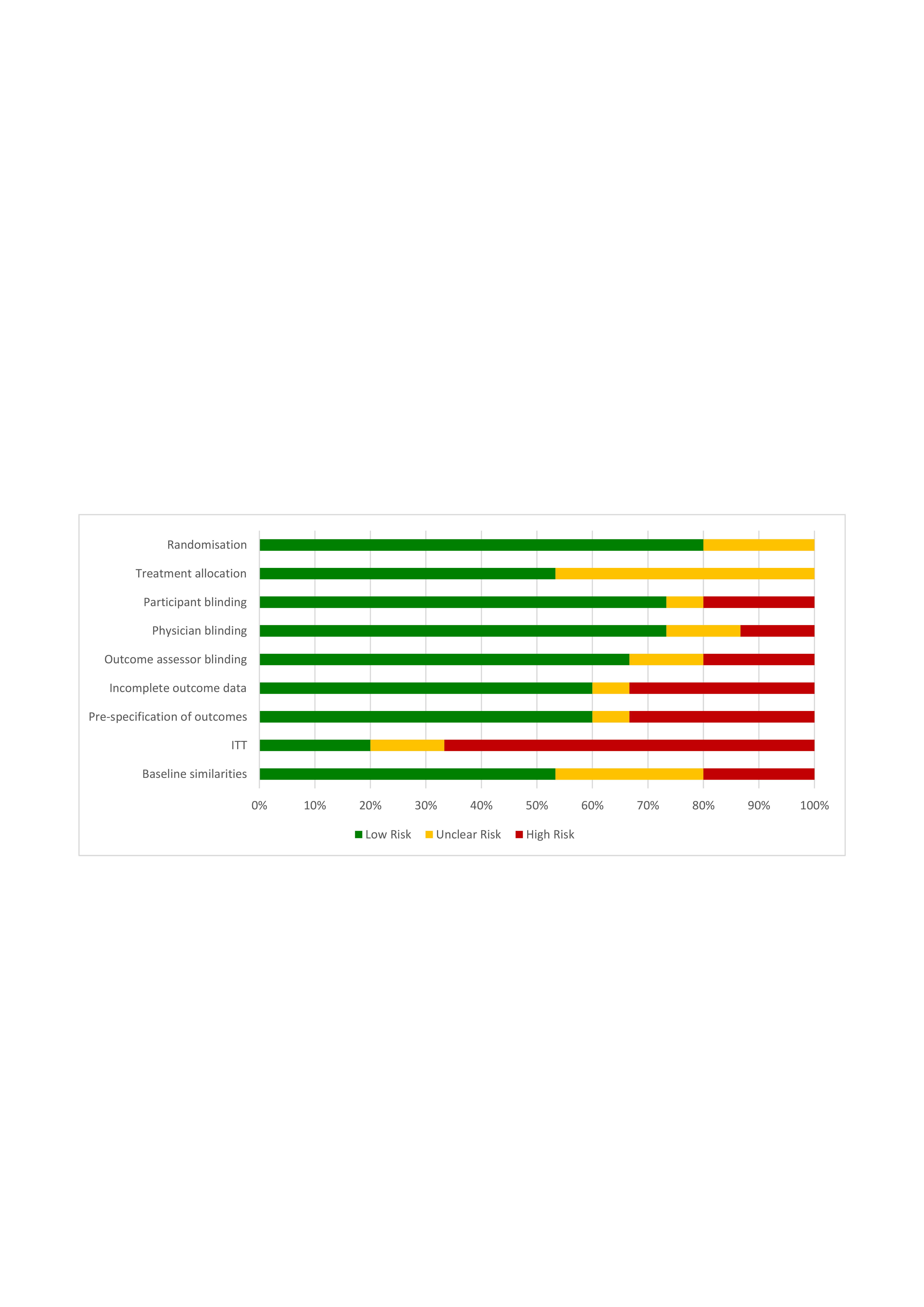

Supplement: keaa113_Supplementary_Data [file keaa113_supplementary_data.zip › keaa113-Suppl_Data/rhe-19-1496-File012.tiff]

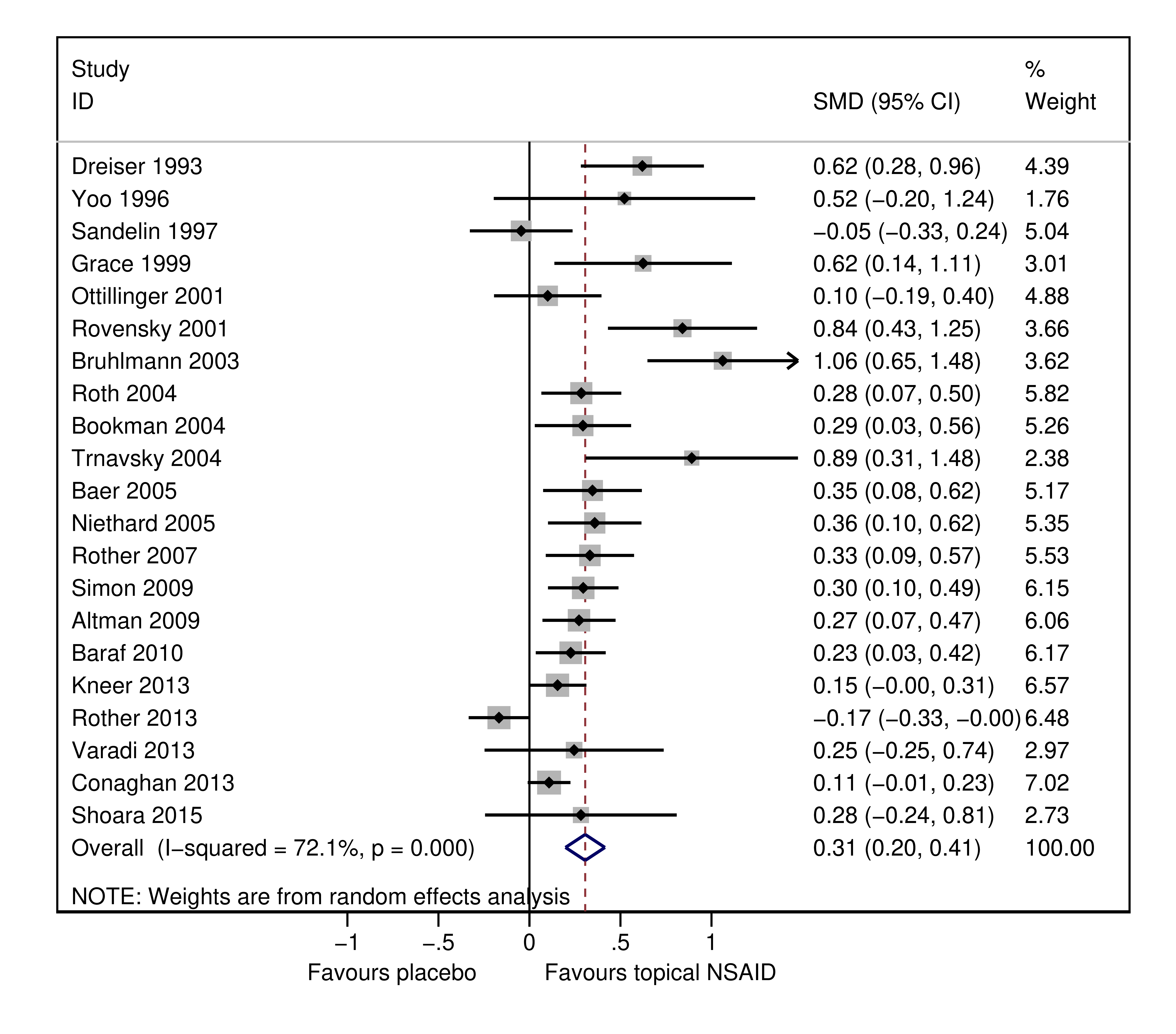

Supplement: keaa113_Supplementary_Data [file keaa113_supplementary_data.zip › keaa113-Suppl_Data/rhe-19-1496-File009.tiff]

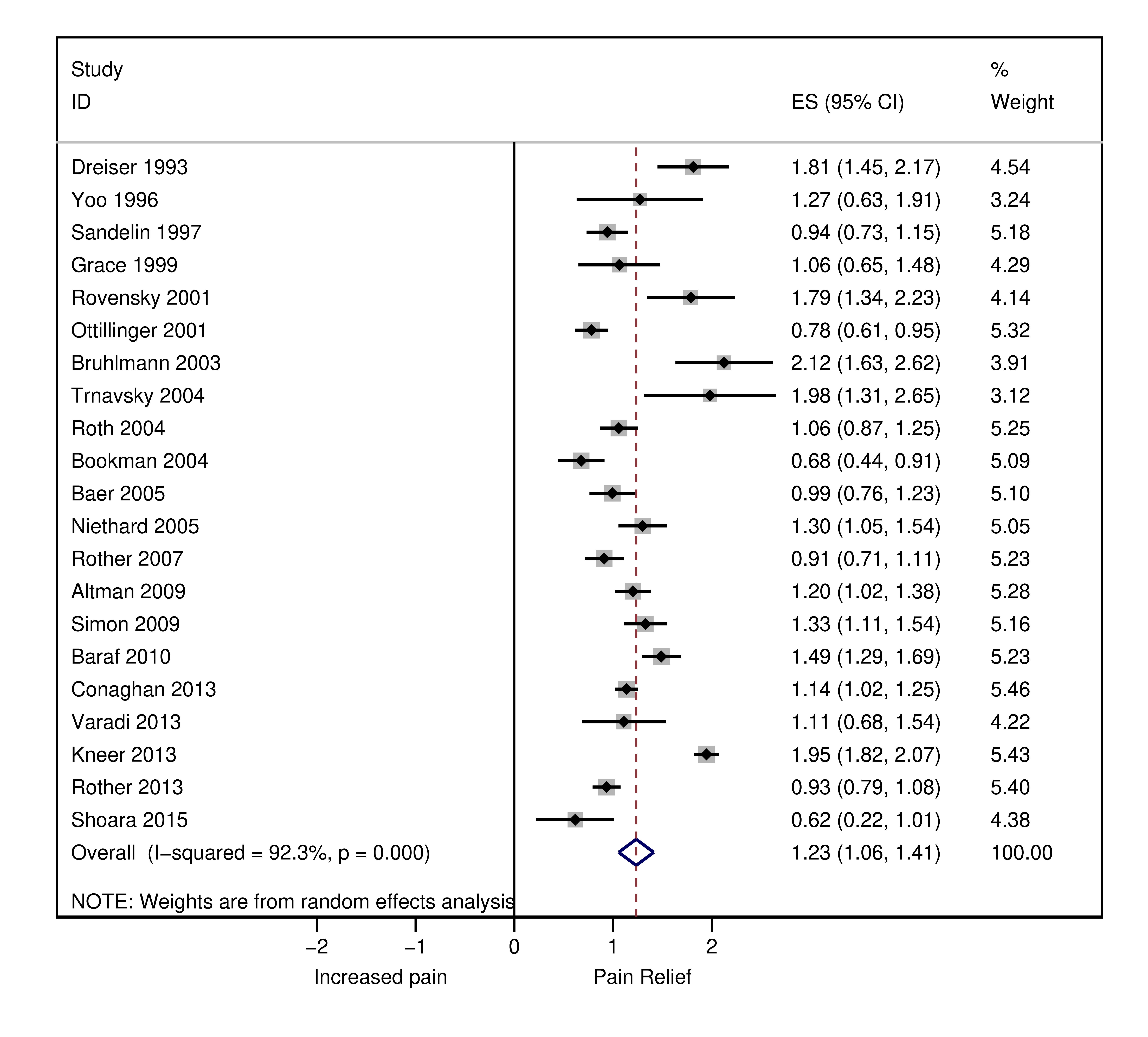

Supplement: keaa113_Supplementary_Data [file keaa113_supplementary_data.zip › keaa113-Suppl_Data/rhe-19-1496-File010.tiff]

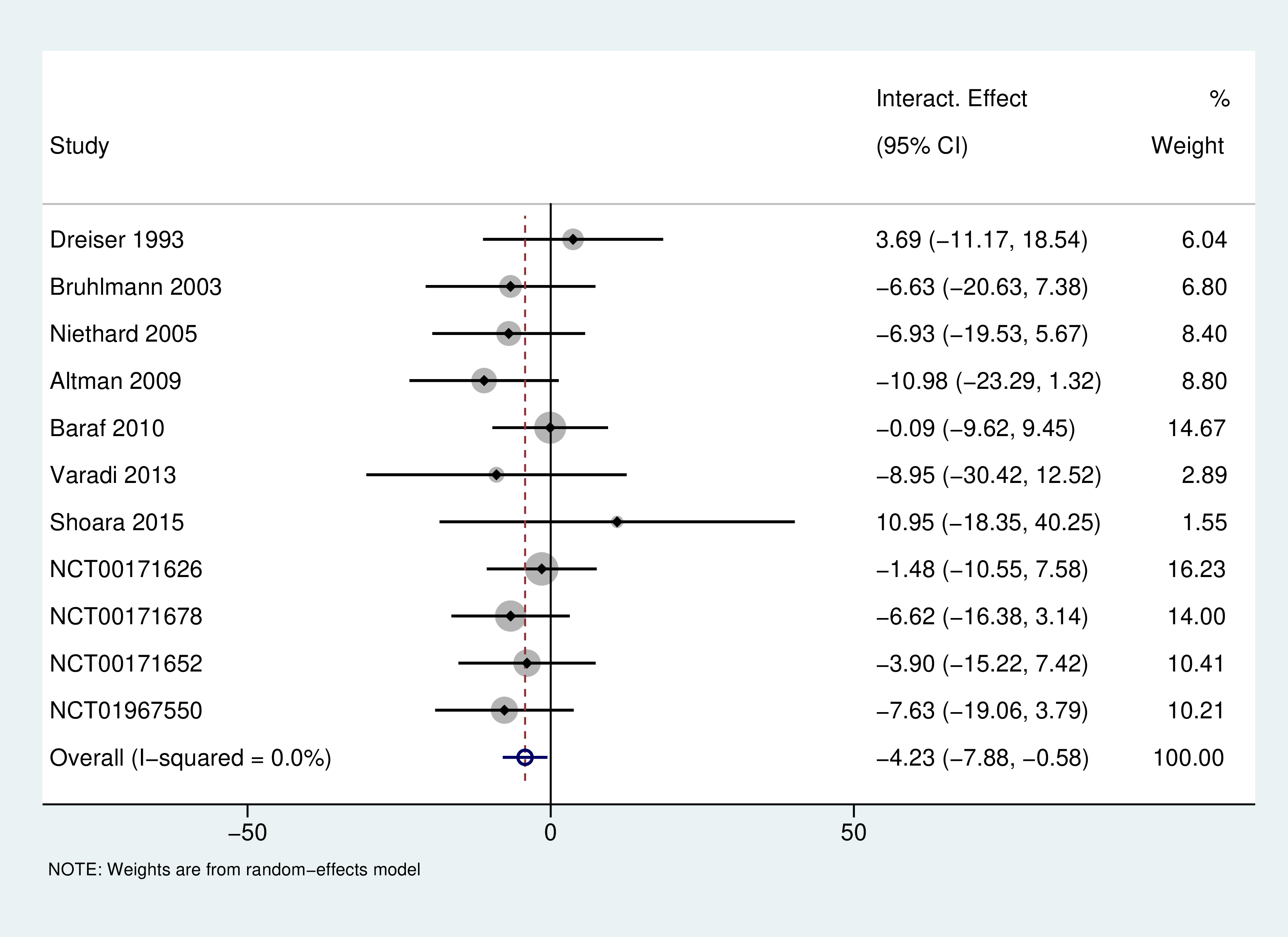

Supplement: keaa113_Supplementary_Data [file keaa113_supplementary_data.zip › keaa113-Suppl_Data/rhe-19-1496-File011.tiff]
